# Acute Obstructive Suppurative Pancreatic Ductitis in an Asymptomatic Patient

**DOI:** 10.1155/2015/919452

**Published:** 2015-01-20

**Authors:** Eisha Wali, Patrick Koo, Clifford D. Packer

**Affiliations:** ^1^Department of Internal Medicine, Louis Stokes Cleveland VA Medical Center, Cleveland, OH 44106, USA; ^2^Case Western Reserve University School of Medicine, Cleveland, OH 44106, USA; ^3^Department of Internal Medicine, Case Western Reserve University and University Hospitals Case Medical Center, Cleveland, OH 44106, USA

## Abstract

Acute obstructive suppurative pancreatic ductitis (AOSPD), defined as suppuration from the pancreatic duct without associated pseudocyst, abscess, or necrosis, is a rare complication of chronic pancreatitis. We present the first case of AOSPD in an asymptomatic patient with a polymicrobial infection and review the literature on this rare clinical entity.

## 1. Introduction

Acute obstructive suppurative pancreatic ductitis (AOSPD) is a rare complication of chronic pancreatitis that has been described in only six previous case reports [1–6]. AOSPD is defined as suppuration from the pancreatic duct; however, in contrast to the pancreatic infections that typically complicate chronic pancreatitis, it is not associated with pancreatic pseudocyst, abscess, or necrosis.

In prior cases of AOSPD, patients presented with fever, abdominal pain, and objective signs of infection. We present the first case of AOSPD in an asymptomatic patient without clinical signs of infection. Our case is also unique as it is the first case involving a polymicrobial infection, with the majority of pathogens likely originating from the respiratory tract.

## 2. Case Presentation

A 63-year-old Caucasian man with a history significant for chronic alcoholic pancreatitis, multiple hospitalizations for acute on chronic pancreatitis, and choledocholithiasis status post prior endoscopic retrograde cholangiopancreatography (ERCP) and sphincterotomy presented for elective ERCP for pancreatic ductal dilatation noted on imaging.

**Figure 1:**
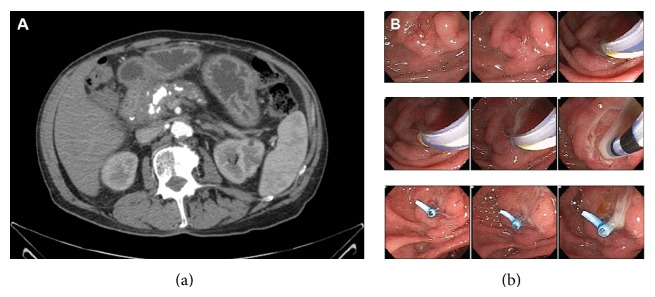
(a) CT scan prior to ERCP, illustrating evidence of chronic pancreatitis and pancreatic duct stricture, without associated pseudocyst, abscess, or necrosis and (b) ERCP demonstrating frank purulent drainage at the pancreatic duct.

One month prior to admission, he was seen in the clinic for management of chronic pancreatitis. During this visit, the patient noted persistent epigastric pain similar to his prior chronic pancreatitis pain. CT abdomen with contrast revealed chronic atrophic calcific pancreatitis and marked main pancreatic duct dilatation to 15 mm (Figure 1(a)). There was no evidence of pancreatic pseudocyst, abscess, or necrosis. He was scheduled for elective ERCP.

Several weeks prior to ERCP, his epigastric pain resolved. He denied fevers or chills. On ERCP, the major papilla of Vater was seen spontaneously expelling pus. Pancreatogram revealed severe irregularity of the head of the pancreas, mild stricture in the neck of the pancreas, and severe main pancreatic duct dilatation to 15 mm. A 7-French 10 cm straight plastic stent was placed in the main pancreatic duct. Following stent placement, a large amount of purulent material was seen draining from the stent (Figure 1(b)). The fluid was collected from the main pancreatic duct via catheter and sent for culture. The patient was admitted for intravenous antibiotics and monitoring.

On admission, he was afebrile and hemodynamically stable. His physical exam and labs were unremarkable. Notably, his white blood cell count was 6.04 × 10^9^/L. CT showed resolution of main pancreatic duct dilatation and was otherwise unchanged. He was empirically started on Vancomycin and Ertapenem.

Cultures of the purulent pancreatic duct fluid were positive for* Escherichia coli, Streptococcus pneumoniae,* and* Haemophilus influenzae*, all sensitive to fluoroquinolones. He remained afebrile throughout his hospital stay and was ultimately transitioned to oral moxifloxacin. Following discharge, he remained asymptomatic. ERCP was repeated 6 days after admission for stent exchange and revealed a small amount of purulent drainage following replacement. Repeat ERCP 1 month later noted main pancreatic duct dilatation and numerous pancreatic duct stones. The stones were removed and his pancreatic duct stent was upsized. There was no evidence of purulent drainage. Two months later, he underwent his final ERCP and his stent was extracted and not replaced.

## 3. Discussion

This case of acute obstructive suppurative pancreatic ductitis (AOSPD) represents the first reported case in an asymptomatic patient without any clinical signs of infection. A MEDLINE search revealed six prior cases of AOSPD (Table 1) [1–6].

The presentation and severity of illness of AOSPD can vary significantly. In prior cases, patients presented with abdominal pain, with severity of illness ranging from meeting criteria for systemic inflammatory response syndrome (SIRS) [1, 2, 4] to septic shock [3]. In contrast, our patient was asymptomatic and had no objective signs of infection at the time of diagnosis and throughout his hospitalization.

Our case is also unique because of the polymicrobial nature of the infection. Prior cases were monomicrobial [1, 3–5]. Two of the three organisms isolated in our case,* Streptococcus pneumoniae* and* Haemophilus influenza*, are generally considered respiratory pathogens [7, 8]. However, both have also been associated with other complications of pancreatitis, including pancreatic abscess and pseudocyst [9, 10].

Several theories to explain how respiratory pathogens may infect the pancreas have been proposed. Possible hypotheses include hematogenous or lymphatic spread from nasopharyngeal colonization, enteric spread from transient inclusion of the bacteria in intestinal flora, or the direct introduction of nasopharyngeal flora into the biliary tree and pancreatic duct during endoscopic interventions such as ERCP [9]. ERCP is a well-known risk factor for cholangitis through the introduction of bacteria to the biliary tree and may seed infection in AOSPD through a similar mechanism [2, 9, 10].

While the pathogenesis of AOSPD is not completely understood, chronic pancreatitis [2, 3], prior sphincterotomy [1], pancreatic stasis secondary to pancreatic duct obstruction [1, 2], and diabetes mellitus [1, 3] have all been implicated as possible risk factors for its development. All of these potential risk factors were present in our patient. Theoretically, sphincterotomy may allow for reflux of bacteria from the gastrointestinal tract or biliary tree into the pancreatic duct and pancreatic stasis encourages uncontrolled proliferation of bacteria. Diabetes mellitus creates a general predisposition to infection, likely stemming from impairments in neutrophil chemotaxis and adherence to vascular endothelium, phagocytosis, and cell mediated immunity [11, 12]. Other causes of immune dysfunction, while not present in our patient, have also been associated with AOSPD. Fujimori et al. reported a case of AOSPD in the setting of peripheral blood stem cell transplantation for acute myeloid leukemia and subsequent chronic leucopenia [4]. Tajima et al. described AOSPD in the setting of pancreatic cancer; although carcinoma of the head of the pancreas does not inherently cause an immunocompromised state as seen with some hematologic malignancies, it can predispose to infection from biliary or pancreatic obstruction [2, 13]. Furthermore, impaired antibacterial activity of pancreatic secretions in the setting of chronic pancreatitis may also contribute to development of AOSPD [2, 3].

In all reported cases of AOSPD, diagnosis was made by identification of purulence at the main pancreatic duct on ERCP in the absence of evidence of other pathologies, such as pancreatic pseudocyst or abscess. Only one patient in prior published AOSPD case reports received an MRCP prior to ERCP [2]. This was done in the setting of a carcinoma of the head of the pancreas, and MRCP revealed a dilated pancreatic duct but no evidence of infection in the duct.

Once diagnosed, the mainstay of treatment is prompt intravenous antibiotics and pancreatic duct decompression and drainage [1–6]. AOSPD appears to respond quickly and dramatically to this treatment.

It remains unclear why AOSPD is such a rare clinical entity while cholangitis is much more common. Chronic pancreatitis, pancreatic ductal abnormalities including stricture or obstruction, and ERCP are all relatively common. However, only six cases of AOSPD have been described since 1995, suggesting there likely are other factors contributing to its pathogenesis. While AOSPD is a rare complication of chronic pancreatitis, physicians should keep AOSPD on their differential diagnosis in patients with chronic pancreatitis and pancreatic ductal dilatation, even in the absence of symptoms or signs of infection. Future case reports will hopefully continue to provide more information about this rare clinical entity.

## Figures and Tables

**Table 1:** AOSPD case reports.

Case report	Patient age/gender	Prior endoscopic intervention	Relevant comorbid conditions	Presenting symptoms	WBC	CT scan results	ERCP results	Culture results	Clinical course
Weinman [1]	74/male	Biliary sphincterotomy	Chronic pancreatitis, DM	Abd. pain, fever, N/V	17.9	Dilated pancreatic duct with 5 mm stone	Pancreatic duct stone removed, stented	*E. coli *	Resolved, doing well at 18 months

Tajima et al. [2]	73/male	No	Chronic pancreatitis, pancreatic CA	Abd. pain, fever	14.2	Dilated pancreatic duct, tumor at head of pancreas	Pancreatic stricture, stented	N/A	Resolved

Deeb et al. [3]	46/male	ERCP, pancreatography	Chronic pancreatitis	Abd. pain, fever	N/A	Dilated pancreatic duct with large stone	Pancreatic duct stone, stented	*Klebsiella ornithinolytica *	Resolved

Fujimori et al. [4]	53/male	No	Chronic pancreatitis, AML	Abd. pain, fever	3.19	Dilated pancreatic duct with stones	Pancreatic duct cannulated and stented	*Stenotrophomonas maltophilia *	Relapsed one month later and required repeat ERCP and drainage

Fujinaga et al. [5]	70/male	No	Intraductal mucinous neoplasm	Abd. pain, fever	N/A	Mild pancreatic edema, 10 mm pancreatic stone	Pancreatic duct cannulated	*Klebsiella oxytoca *	Resolved

Aoki et al. [6]	50/male	N/A	Chronic pancreatitis	Abd. pain, fever	N/A	N/A	Purulent pancreatic fluid, main pancreatic duct stented	N/A	Resolved

Wali et al. (this case)	63/male	Biliary sphincterotomy	Chronic pancreatitis, DM	Asymptomatic	6.04	Dilated pancreatic duct, pancreatic calcifications	Pancreatic stricture, stented	*E. coli, S. pneumoniae,* and *H. pneumoniae *	Resolved

## Abbreviations

AOSPD: Acute obstructive suppurative pancreatic ductitis
ERCP: Endoscopic retrograde cholangiopancreatography.
